# Shared decision making in elderly patients with kidney failure

**DOI:** 10.1093/ndt/gfad211

**Published:** 2023-09-23

**Authors:** Mehmet Kanbay, Carlo Basile, Yuri Battaglia, Alessandro Mantovani, Furkan Yavuz, Francesco Pizzarelli, Valerie A Luyckx, Adrian Covic, Vassilios Liakopoulos, Sandip Mitra

**Affiliations:** Division of Nephrology, Department of Medicine, Koc University School of Medicine, Istanbul, Turkey; Associazione Nefrologica Gabriella Sebastio, Martina Franca, Italy; Department of Medicine, University of Verona, Verona, Italy; Nephrology and Dialysis Unit, Pederzoli Hospital, Peschiera del Garda, Verona, Italy; Section of Endocrinology, Diabetes and Metabolism, Department of Medicine, University and Azienda Ospedaliera Universitaria Integrata of Verona, Verona, Italy; Division of Nephrology, Department of Medicine, Koc University School of Medicine, Istanbul, Turkey; Past Director, Nephrology Unit, SM Annunziata Hospital, Florence, Italy; Renal Division, Brigham and Women's Hospital, Harvard Medical School, Boston, MA, USA; Department of Paediatrics and Child Health, University of Cape Town, South Africa; Department of Public and Global Health, Epidemiology, Biostatistics and Prevention Institute, University of Zurich, Zurich, Switzerland; Nephrology Clinic, Dialysis and Renal Transplant Center – ‘C.I. Parhon’ University Hospital, and ‘Grigore T. Popa’ University of Medicine, Iasi, Romania; Second Department of Nephrology, AHEPA University Hospital, Medical School, Aristotle University of Thessaloniki, Thessaloniki, Greece; Manchester Academy of Health Sciences Centre (MAHSC), Manchester University Hospitals and University of Manchester, Manchester, UK

**Keywords:** elderly patients, frailty, hemodialysis, peritoneal dialysis, vascular access

## Abstract

‘Elderly’ is most commonly defined as an individual aged 65 years or older. However, this definition fails to account for the differences in genetics, lifestyle and overall health that contribute to significant heterogeneity among the elderly beyond chronological age. As the world population continues to age, the prevalence of chronic diseases, including chronic kidney disease (CKD), is increasing and CKD frequently progresses to kidney failure. Moreover, frailty represents a multidimensional clinical entity highly prevalent in this population, which needs to be adequately assessed to inform and support medical decisions. Selecting the optimal treatment pathway for the elderly and frail kidney failure population, be it haemodialysis, peritoneal dialysis or conservative kidney management, is complex because of the presence of comorbidities associated with low survival rates and impaired quality of life. Management of these patients should involve a multidisciplinary approach including doctors from various specialties, nurses, psychologists, dieticians and physiotherapists. Studies are mostly retrospective and observational, lacking adjustment for confounders or addressing selection and indication biases, making it difficult to use these data to guide treatment decisions. Throughout this review we discuss the difficulty of making a one-size-fits-all recommendation for the clinical needs of older patients with kidney failure. We advocate that a research agenda for optimization of the critical issues we present in this review be implemented. We recommend prospective studies that address these issues, and systematic reviews incorporating the complementary evidence of both observational and interventional studies. Furthermore, we strongly support a shared decision-making process matching evidence with patient preferences to ensure that individualized choices are made regarding dialysis vs conservative kidney management, dialysis modality and optimal vascular access.

## INTRODUCTION

The measurement of aging is commonly based on chronological age, with individuals aged 65 years or older often referred to as ‘elderly’. However, the aging process is not uniform across the population due to variations in genetics, lifestyle and overall health. Therefore, using chronological age alone fails to capture the significant heterogeneity observed within the elderly population. Considering the current life expectancy of 80 years or more in some high-income countries, there is a need to revisit the definition of ‘elderly’ based on comprehensive evidence encompassing social, cultural and medical factors [1]. The world's population is experiencing an increase in aging. The median age has risen in the last 40 years, and projections for global demographics suggest that the number of people aged 65 years or more will triple over the coming decades, reaching over 1.3 billion by 2040 [2, 3]. Alongside the aging population, the prevalence of chronic conditions such as hypertension, diabetes mellitus and chronic kidney disease (CKD) is also increasing globally [2, 3]. CKD, defined as glomerular filtration rate (GFR) <60 mL/min/1.73 m^2^, is common and affects approximately 1 in 10 adults, with the prevalence rising to 1 in 6 among the elderly. It may progress to end-stage kidney failure [4, 5]. A systematic analysis of worldwide population-based data on the global burden of CKD in 2010 demonstrated an age-dependent increase in the prevalence of CKD across all stages [6]. Women exhibited a higher prevalence of CKD than men in middle-age groups, and this sex difference became more pronounced in older age groups, particularly for stages 3–5, although fewer women were receiving kidney replacement therapy (KRT) for reasons not yet fully understood [6]. The number of patients over 65 years of age diagnosed with kidney failure and requiring KRT is steadily increasing [7]. According to the 2015 Annual Report of the European Renal Association–European Dialysis and Transplantation Association (ERA-EDTA) registry, 42% of the total KRT population in Europe was over 65 years old [8]. Similarly, the Annual Data Report published by the United States Renal Data System in 2022 reported the highest rates of treated kidney failure in the elderly population over 75 years of age, at 1447 per million population [9].

Given the growing number of older patients with kidney failure, it is crucial to consider carefully appropriate management strategies in this population, taking into account the effects of aging, comorbidities and frailty, as well as the advantages and drawbacks of various treatment options [10].

This narrative review aims to address crucial issues in the management of elderly patients with kidney failure: (i) the choice between dialysis and conservative kidney management (CKM); (ii) if dialysis is chosen, the selection of specific dialysis modalities, namely haemodialysis (HD) or peritoneal dialysis (PD); (iii) if HD is chosen, the most appropriate type of vascular access (VA); and (iv) when faced with these three management decisions, the use of a shared decision making (SDM) approach.

## SEARCH STRATEGY AND SELECTION CRITERIA

We conducted a comprehensive search of the Cochrane Library, MEDLINE (via Scopus), Embase and PubMed databases for articles published from 1 January 2002 up to 28 February 2023 in English language. However, we did not exclude relevant and highly referenced older publications.

We used the following search terms: ‘conservative kidney management’, ‘conservative treatment’, ‘elderly’, ‘older’, ‘quality of life’, ‘chronic kidney disease’, ‘end-stage kidney disease’, ‘end-stage renal disease’, ‘kidney failure’, ‘renal replacement therapy’, ‘dialysis’, ‘hemodialysis’, ‘peritoneal dialysis’, ‘continuous ambulatory peritoneal dialysis’, ‘vascular access’, ‘central venous catheter’, ‘arteriovenous fistula’ and ‘arteriovenous graft’.

The selection process involved a qualitative assessment of relevant literature to provide an overview and critical evaluation of the available evidence. We carefully examined the titles and abstracts of all identified studies to assess their relevance to our research questions. The criteria for selecting related and unrelated studies were based on their alignment with the scope of our review, their contribution to the topic's understanding and the quality of evidence presented. Furthermore, we constructed supplementary tables summarizing the main findings of the most relevant studies addressing the critical issues in the management of elderly patients with kidney failure described above.

## WHICH CHOICE BETWEEN CKM AND DIALYSIS?

Elderly patients with kidney failure often have multiple comorbidities and are frequently frail, which makes them unsuitable candidates for kidney transplantation. As a result, only two treatment options are available: CKM and dialysis [11–13]. CKM provides conservative and patient-centred individualized medical care to those who opt not to undergo dialysis. The main goals of CKM are to optimize quality of life (QoL) through ongoing medical management, symptom control and advanced care planning (ACP) [14].

In comparison with CKM, dialysis may prolong life [15], but this potential benefit may come at the cost of QoL. The tradeoff between quantity and QoL for each patient is nuanced and requires careful consideration [15–19]. Interestingly, in an analysis of 22 cohort studies, Voorend *et al*. showed that patients opting for dialysis were generally younger and had fewer comorbid conditions, functional impairments and frailty than those who chose CKM [19]. The unadjusted median survival ranged from 20 to 67 months for dialysis and from 6 to 31 months for CKM [19]. A meta-analysis of 12 studies showed a pooled adjusted hazard ratio (aHR) of 0.47 for mortality in patients choosing dialysis compared with CKM. Even in subgroups of patients with older age or severe comorbidities, the reduction in mortality risk in HD vs CKM remained statistically significant, although the analyses were unadjusted [19]. However, in a retrospective cohort, especially among patients with multiple comorbidities, the survival advantage of dialysis over CKM was diminished [20]. Furthermore, several studies have shown a reduced risk of hospitalization among elderly patients receiving CKM compared with dialysis [15, 17, 21].

Survival is not the sole consideration; health-related quality of life (HRQoL) and lifestyle-related outcomes are equally important [22]. The ongoing European Quality (EQUAL) study involving 456 patients aged over 65 years found that symptom burden was significantly higher in the year before dialysis initiation but stabilized after dialysis initiation, although fatigue, decreased interest in sex and sexual arousal remained the most burdensome symptoms [23]. A meta-analysis indicated that CKM may provide a benefit in terms of QoL [11]. Furthermore, a cross-sectional study conducted in the UK and Australia demonstrated that elderly patients on dialysis experienced lower QoL due to symptom burden compared with CKM [24]. However, another recent observational study with 604 patients showed no significant QoL changes or difference in symptom burden between CKM and dialysis groups [25]. In another cohort with a mean age of 64.0 ± 10.5 years, lower executive function was observed during transition to dialysis [26].

Two ongoing studies hold promise. The first is the DIALysis or not: Outcomes in older kidney patients with GerIatriC Assessment (DIALOGICA) study, a prospective, observational cohort study planned to enroll 1500 patients from 25 Dutch and Belgian centres. It aimed to compare HRQoL, clinical outcomes and costs between CKM and dialysis in older patients. The total follow-up will be a maximum of 4 years. By generating more insights into the impact of CKM and dialysis on HRQoL, clinical outcomes and costs, this study will support patients and physicians to reach informed shared decisions on the best individual treatment option for kidney failure [27]. The second study is a randomized controlled trial (RCT) called the Prepare for Kidney Care Study, which aims to compare the Quality-Adjusted Life Years (QALYs) gained after 3 years of dialysis and CKM in older patients with CKD. This study will provide further insights into best practices in this field by considering person-centred outcomes and providing unbiased information in support of SDM [28].

Supplementary data, Table S1 summarizes the main findings of the most relevant studies comparing dialysis with CKM in elderly patients [15–18, 20, 21, 24–26]. It is important to note that these studies are mostly retrospective and observational, lacking adjustment for confounders or address selection and indication biases, making it difficult to use these data to guide treatment decisions. Interpreting the currently available evidence regarding dialysis vs CKM among elderly patients in terms of survival, QoL and other clinical outcomes is challenging and requires further high-quality studies to support SDM.

### Nutritional support in elderly patients with kidney failure

These patients are particularly vulnerable to nutritional deficiencies, such as protein-energy wasting and fluid or electrolyte imbalances. Over time, they experience a gradual decline in their nutrition status, with depletion of protein and energy reserves, often due to dietary restrictions, gastrointestinal problems or low socioeconomic status. These factors lead to increased morbidity and mortality, and a decreased QoL [29]. The optimal nutrition plan should be individualized considering the patient's treatment goals. Several nutrition tools, like the Malnutrition Inflammation Score [30] or the Integrative Clinical Nutrition Dialysis Score [31], may be used to detect malnutrition early and initiate prompt intervention.

According to the Kidney Disease Outcomes Quality Initiative (KDOQI) Clinical Practice Guidelines, for patients with CKD stages 3–5 not on dialysis, a low protein intake (0.55–0.60 g/kg/day) or very low protein intake (0.28–0.43 g/kg/day) with additional keto-acid analogues, and 0.6–0.8 g/kg/day for those with diabetes, under close clinical supervision, is suggested to reduce the risk of end-stage kidney failure and improve QoL [32]. Recently, the European Society for Clinical Nutrition and Metabolism (ESPEN) and the European Renal Nutrition group of the European Renal Association (ERN-ERA) jointly published for the first time a critical review paper regarding the optimal protein and energy intake for older adults with varying degrees of CKD severity. This critical review aimed to address some significant questions. Among them: is there any evidence supporting the effectiveness of low-protein diets in older patients with CKD? The authors concluded that not all older adults with CKD require dietary modifications with the implementation of a low-protein diet. In fact, those with stable or slowly progressing CKD, especially those at early stages of the disease, may not derive any benefits from a low-protein diet, as they will probably never reach kidney failure. At the opposite end of the scale, patients with or at high risk of malnutrition, with impairment in functional status or with a limited life expectancy, also may not benefit from a low protein diet [33].

On the other hand, for patients undergoing HD or PD a higher daily protein intake is suggested due to protein loss into the dialysis solution and the presence of ongoing inflammatory stimuli [32–34]. In elderly patients who are more fragile compared with the general population, reducing protein intake may lead to protein wasting. Furthermore, it is important to maintain electrolyte balance and prevent fluid overload in patients with kidney failure. Dietary sodium and potassium restrictions, along with vitamin D supplementation and phosphate restriction, are suggested to prevent fluid overload, hyperkalemia and calcium-phosphate balance disorders [35]. If dietary counselling fails to improve patients’ nutrition status, oral nutritional supplements can be considered as effective alternatives for restoring protein and energy resources. If these measures do not yield the desired results, more invasive approaches like enteral tube feeding, intraperitoneal or intradialytic parenteral nutrition, should be considered [29]. However, it is important to acknowledge that parenteral nutrition is an invasive and costly method of providing nutrition support, which also carries an increased risk of metabolic and septic complications [29]. Moreover, transitioning to enteral feeding or other forms of feeding may not always be appropriate or desired by patients or their caregivers. This highlights the importance of SDM in addressing such issues.

## IF DIALYSIS IS CHOSEN, WHICH MODALITY TO SELECT?

The next step is selecting the most appropriate dialysis modality. All dialysis modalities may affect survival, QoL and neurocognitive outcomes. However, despite numerous studies, there is no clear indication as to which dialysis modality is most suitable for the elderly population.

In-centre HD may be convenient for elderly patients as it provides continuous follow-up and an opportunity for social interaction [36]. However, it is crucial not to overlook the associated complications, such as the risk of hypotension during dialysis, infections, gastrointestinal bleeding and malnutrition [37]. For some elderly individuals the stress of adhering to an early morning shift, the dependence on potentially unreliable transport and the lack of flexibility of in-centre dialysis can result in disrupted sleep. In addition, the fear of complications such as hypotension, feeling cold and cramps can contribute to significant stress associated with in-centre HD.

The pattern of dialysis initiation whether on PD or HD affects clinical outcomes, as unplanned start of dialysis has been linked to poorer outcomes compared with planned start of dialysis [38]. Of note, urgent-start PD strategies have been shown to be safe and associated with fewer complications in the first 6 weeks after dialysis initiation compared with urgent-start HD strategies [39]. In terms of mortality, some studies have demonstrated that HD provides better outcomes compared with PD [40, 41]. However, more recent studies indicated that HD and PD were associated with similar mortality rates among incident dialysis patients who were eligible for both modalities [42]. Nevertheless, as a result of multiple comorbidities and fewer functional and cognitive capacities, older age may be considered a relative contraindication for PD [43]. Nonetheless, PD does offer advantages, including the potential for better QoL, higher satisfaction [44, 45], improved cardiovascular stability [37], reduced travel frequency to dialysis centres and increased autonomy [46]. It should be noted that assuming sole responsibility for home dialysis can be intimidating for some individuals. Fortunately, advancements in automated PD (APD) and continuous ambulatory PD (CAPD) have made PD more accessible for many elderly patients who were previously not considered candidates for PD. For frail patients, the physical strength required to handle compartmentalized dialysis bags may pose an additional barrier, but assistance and support from visiting nurses or caregivers can help overcome these obstacles, particularly with APD [40].

Most data comparing HD with PD in older patients come from observational studies, which report varying outcomes in terms of mortality and morbidity. As highlighted in a meta-analysis by Han *et al*., during the first year of dialysis there was no difference in outcomes between HD and PD in the elderly, but after 1 year, PD patients had a higher mortality rate (HR 1.42, *P* < .001) [47]. Moreover, some studies demonstrated that diabetes is an important factor contributing to mortality in PD patients. Specifically, PD patients with diabetes have been found to have worse survival rates [48].

Consensus regarding the impacts of HD vs PD on survival and QOL has not been reached. A recent study suggested comparable outcomes between PD and HD in terms of QoL [45], while another one showed that patients receiving PD had worse cognitive dysfunction and worse HRQoL compared with patients receiving HD [49]. A prospective cohort study of 174 patients older than 70 years found similar QoL between PD and HD patients [50]. Recently, a meta-analysis by Chuasuwan *et al*. found that HRQoL was better in patients on PD compared with HD [51]. Accordingly, a further systematic review and meta-analysis showed that patients treated with PD have better cognitive outcomes and a lower risk of dementia [52]. Accidental falls were found to be equally common in patients receiving PD compared with HD (odds ratio 1.63, *P* = .1) [53].

It is important to plan dialysis as early as possible [54] and any obstacles to dialysis should be assessed by a team of healthcare professionals, who can then address them by providing adequate care and information, as well as social and psychological support.

Supplementary data, Table S2 summarizes the main findings of the most relevant studies comparing PD with HD in elderly patients [39–43, 45, 49, 50, 53, 55].

Incremental dialysis, which is an alternative approach to dialysis, be it PD or HD, may be a suitable option, especially for frail patient groups [56, 57]. This approach allows a gradual and smoother transition to dialysis, minimizing the disruptive impact on daily life [58]. A meta-analysis of 22 observational studies reported that incremental dialysis resulted in a lower mean loss of residual kidney function (–0.58 mL/min/month, *P* = .007) than a full-dose start [59]. Additionally, another meta-analysis showed no significant difference in mortality between the incremental and conventional HD groups (HR 0.99; I^2^ = 82%) [60]. Recently, an RCT, albeit with limited sample size, also found that incremental dialysis was associated with a 69% lower risk of hospitalization compared with full-dose dialysis [61]. However, it is important to note that these studies were not specifically designed for geriatric patients. Therefore, more RCTs with larger and older patient groups, as well as observational studies, are needed to further investigate this approach.

## IF HD IS CHOSEN, WHICH TYPE OF VA TO ADOPT?

A durable VA is required for life-sustaining HD. Its successful creation and maintenance are crucial for patients who rely on regular HD [62, 63]. An ideal VA should have long-term durability with a low rate of complications, while ensuring adequate blood-flow rate to deliver the recommended dialysis dose [64]. Despite the lack of RCTs specifically investigating the superiority of VA types in elderly patients, the choice of a VA is associated with variable patient morbidity and mortality [64].

### Arteriovenous fistulas and arteriovenous grafts

Traditionally, arteriovenous fistulas (AVF) have been considered the first-line choice due to lower infection risk and better long-term patency. However, the recent KDOQI Clinical Practice Guidelines suggested a patient-centred and individualized approach when planning a VA, considering a patient's preferences and goals (i.e. ‘the right access, in the right patient, at the right time, for the right reasons’) [65]. This approach is particularly relevant for elderly patients with multiple comorbidities, as the AVF-first strategy should be carefully evaluated considering their lower life expectancy, longer maturation time of the VA and risk of patency loss [66–68]. Nevertheless, several studies have shown that the construction of an AVF in elderly patients is still technically feasible and that AVF may the preferred VA option, as it is associated with fewer complications [64, 69]. For instance, among elderly incident HD patients who initiated dialysis with a central venous catheter (CVC), those who subsequently received an AVF within 6 months had fewer hospitalizations and infections compared with those who received an arteriovenous graft (AVG) [70].

The timing of VA creation remains a crucial question. Given the shorter life expectancy of elderly individuals, many AVFs established before HD initiation may never be utilized. Among a cohort of 3418 elderly patients who underwent pre-dialysis VA creation, 67.4% started dialysis, 15.1% died and 17.5% survived without requiring dialysis by the end of the follow-up period [71]. In addition, patients with a life expectancy of less than 18 months may not benefit from the prolonged patency offered by an AVF compared with an AVG, as the advantages of AVFs over AVGs become apparent only after 18 months [72]. Liu *et al*. studied 184 early elderly patients (aged 65–75 years) and 86 late elderly patients (above 75 years) and noted that more interventions were required in late elderly patients due to the longer maturation time of AVFs [73]. Two recent studies demonstrated AVF primary failure rates of 22.1% and 27%, respectively, in patients over 75 years old [73, 74], and in another study involving patients in their 80s with a higher diabetes prevalence, the primary AVF failure rate was 72% [75]. Distal AVFs had a higher primary failure rate compared with proximal AVFs [66, 76, 77]. In a retrospective cohort of 941 adult patients, proximal AVFs demonstrated higher patency rates than distal AVFs, with rates of 40 ± 7% and 18 ± 5%, respectively (*P* = .007) [78]. The increased incidence of primary failure in distal AVFs may be related to lower blood-flow rates [79]. On the other hand, proximal AVFs, with their higher access blood flow rates, pose a higher risk of steal syndrome and of high-output heart failure [80].

AVGs offer certain advantages in the elderly population including a shorter waiting time between placement and utilization compared with AVFs, as well as a lower infection risk compared with CVCs [64]. Consequently, some studies suggest that AVGs may be a reasonable first-line choice for elderly patients, considering lower primary failure rates than for AVFs [81]. In addition, Cui *et al*. found that the time to catheter-free dialysis was shorter with AVGs than with AVFs (*P* < .001), and the assisted maturation rate was lower for AVGs (10%) than for AVFs (31%) [82]. However, it should be noted that AVGs can be more costly, necessitate more maintenance interventions and may have greater detrimental effects on the cardiovascular system [64].

### Tunnelled CVCs

CVCs are a viable option for elderly patients as they do not require any maturation time and can be easily and quickly inserted [83]. Additionally, they have fewer bleeding and bruising complications, do not increase cardiac load and do not cause pain during their use [64, 84]. However, in terms of mortality and morbidity, CVCs may not be the best choice. CVCs are associated with higher mortality rates along with higher infection risks compared with AVFs and AVGs [85–88]. A retrospective analysis of 124 421 patients older than 75 years reported that those with CVCs had higher mortality rates compared with those with AVFs (aHR 2.23; *P* < .001) [88]. Another study found no difference in mortality between elderly patients with AVGs or AVFs, but mortality was significantly higher in patients with CVCs [89]. Initiation of dialysis with a CVC was also associated with a higher mortality rate in elderly patients compared with younger patients [90, 91]. Higher mortality rates with CVCs may, however, be confounded by comorbidities and selection bias, such as frailty and acute starts, rather than CVC access–related complications [92]. CVCs can be a good initial option for a subset of patients over 90 years of age with short life expectancy, limited functional status and multiple comorbidities [64]. CVCs may also be preferred when bleeding complications or cardiac load are significant considerations.

The Standardized Outcomes in Nephrology-Hemodialysis (SONG-HD) consensus workshop identified VA as one of the four core outcome domains in HD. Proposed outcome measures for VA function included ‘uninterrupted use of the access without the need for interventions’ and ‘ability to receive prescribed dialysis’ [93]. Furthermore, in a more recent SONG-HD Initiative, a comprehensive process involving a Delphi survey with 1181 participants (including 220 patients and caregivers and 979 health professionals) from 73 countries was conducted to identify outcome domains that should be reported in clinical trials in HD patients. VA was identified as one of the four critically important outcomes by all stakeholder groups [94].

Current KDOQI Clinical Practice Guidelines, however, do not provide specific recommendations for VA in elderly patient. Nonetheless, there are ongoing clinical trials that are currently recruiting participants to investigate the optimal VA for elderly patients [95–97]. Key factors to be considered include QoL, life expectancy, comorbidities and potential complications associated with each type of VA. While a ‘fistula first’ approach has been advocated within the context of the Fistula First Initiative [98], prioritizing individualization (‘patient first’) is more important for elderly patients [64].

Supplementary data, Table S3 summarizes the main findings of the most relevant studies investigating the outcomes of different VA types in elderly patients [66–69, 72–76, 78, 82, 85–90].

## WHY SDM SHOULD BE THE PREFERRED MANAGEMENT APPROACH

Given the uncertainties in outcomes and the varied value individuals may place on potential outcomes, informed SDM with patients and their families is crucial when determining the most appropriate therapeutic pathway. SDM should consider not only clinical perspectives but also QoL and logistical perspectives [13]. Furthermore, it is important to acknowledge that the decision to proceed with dialysis or CKM also affects caregivers, although definitive evidence is lacking. A study assessing the effects of dialysis in elderly patients on functional status and caregiver burden suggested that greater functional deterioration at 6 months was associated with increased caregiver burden [99]. Conversely, another systematic review showed that CKM was related to higher burden and anxiety in caregivers due to fear of deterioration, death and limited involvement in CKM [100]. In clinical practice SDM is not yet routine and needs further implementation. Shifting from a biomedical to a person-centred approach might facilitate a more effective SDM process. To engage in such SDM, healthcare professionals need to become a skilled companion, being part of the patient's relational context, and learning to ask the right questions about what truly matters to the patient as a person [101]. In this context, two supportive tools can be useful in making the best decision, namely: the Kidney Failure Risk Equation (KFRE), which includes eight readily available clinical variables, predicts progression to kidney failure (defined as progression to the need for dialysis among patients with CKD stages 3–5) [102]; and the Bansal equation, a tool using nine variables, which predicts 5-year mortality in non-fragile older patients with CKD stages 3–5 [103]. Although useful, the applicability to decision making regarding dialysis vs CKM is limited given that this tool is designed for mortality prediction in earlier stages of CKD. Furthermore, KFRE was produced from a cohort of patients of various ages undergoing dialysis, so by its nature its applicability to patients who may opt for a CKM pathway is limited. Moreover, KFRE likely overestimates the risk of kidney failure for older patients related to the competing risk of death [104].

Furthermore, several QoL assessment or decision making tools, developed in accordance with the guidelines, can provide comprehensive information about different dialysis modalities and ensure a high level of treatment agreement [105, 106]. In line with this, a recent RCT evaluated the effectiveness of an interactive, web-based decision aid called Decision-Aid for Renal Therapy (DART) in older adults with CKD [107].

SDM is an ethical imperative in clinical medicine, as it upholds the three core principles of biomedical ethics: respect for patient autonomy, maximizing well-being and minimizing harm. Initiating SDM in a timely manner and sharing relevant information about dialysis options with elderly patients allows them and their family/caregivers to have adequate time to consider and discuss the available options, to prepare them for the associated physical (e.g. the placement of a Tenckoff catheter or VA) and emotional aspects, and to anticipate the potential ‘burdens’ of dialysis or the anxieties associated with CKM. Maximizing well-being and minimizing harm should not be limited to solely prolonging life at all costs, but should also include transparent discussions about patient goals, beliefs and what is important to the individual in terms of QoL. It is also important to emphasize that decisions regarding dialysis or CKM may not be irreversible, and patients have the right to change their decisions or to ‘decide not to decide’, and defer decision-making until an emergency arises. Thus, it is crucial to prevent future decisional regrets and conflicts [108].

## PALLIATIVE CARE

When and how to provide palliative care for elderly patients with kidney failure is of paramount importance and should be incorporated into their treatment plan early on. The goal is to reduce the patients’ symptoms, enhance their QoL, and offer emotional and spiritual support to both the patients and their family/caregivers [109]. Elderly patients often experience nonspecific symptoms that can be challenging to deal with, such as pain, sleep disturbances, digestive problems and emotional issues [10]. The use of tools such as the Palliative Care Outcome Scale-Symptoms Modified for Renal Patients and consultation with a multidisciplinary palliative care team can aid in early symptom recognition and facilitate appropriate treatment [110]. The care plan should prioritize patient preferences and wishes for their final days, including effective communication among the patients, the multidisciplinary team, and their family and caregivers [110]. At this step, implementing ACP, which involves understanding and sharing of values, preferences, and goals for future care and treatment, is crucial for patients and families [111]. Yet, integrating ACP into clinical practice remains an important question. An RCT investigating the role of individualized ACP coaching demonstrated that coached patients were more likely to have a documented advanced directive compared with the control group [112]. Another RCT also showed that monthly palliative care visits in addition to usual nephrology care led to improvement in overall and physical symptoms as well as increased adoption of ACP directives [113]. However, there are barriers to ACP in CKD patients and their families, including timing issues, concerns about patient and family ailments, limited resources and expertise, difficulties in predicting outcomes, and a lack of shared understanding within nephrology departments regarding the integration of ACP [114].

## A CALL TO ACTION

The KFRE [102, 115] and the Bansal equation [103] were endorsed by the 2016 European Renal Best Practice (ERBP) Clinical Practice Guidelines for the management of older patients (>65 years) with CKD [116]. With the warnings raised earlier on, the judicious use of these equations in the elderly, a patient with a high Bansal score, indicating a high mortality risk, would benefit from CKM, focusing on ACP rather than stressing the future need for KRT. The same management approach is suggested for the elderly with low mortality risk, but in a frailty state. On the other hand, for patients with low mortality risk, no frailty and a high risk of progression to kidney failure, the guideline recommends maximizing kidney protection and providing pre-dialysis counseling, including modality selection [116]. The authors strongly agree with the ERBP Clinical Practice Guidelines for the management of older patients (>65 years) with CKD; in particular, the authors suggest adopting the decision tree shown in Fig. 1 of this publication [116]. Based on this premise, both the Bansal score and KFRE have been applied in European cohorts of patients aged ≥65 years with kidney failure [117, 118]. In a Norwegian study enrolling elderly patients with eGFR <45 mL/min/1.73 m^2^, good overall agreement between actual and predicted endpoints for both equations was shown, with higher diagnostic accuracy for KFRE as compared with Bansal score [117]. In two Eastern European cohorts, a risk threshold for the Bansal score was proposed, as the ERBP guideline did not define what constitutes a high risk for mortality outcome [118]. This prospective study highlighted the significant value of incorporating a comprehensive geriatric assessment, including frailty, cognitive performance, functional ability, nutrition and depression, alongside risk stratification scores in the evaluation of older patients with kidney failure [118]. Translating this approach into routine clinical practice could lead to more individualized treatment strategies for the heterogeneous population of older patients with kidney failure.

## CONCLUSIONS

We strongly believe that implementation of SDM is crucial for elderly patients with kidney failure, as it can allow the integration of the available evidence with patient preferences, leading to optimal personalized choices regarding treatment options such as dialysis or CKM, dialysis modality and appropriate VA. By prioritizing patient-centred care and involving patients in the decision-making process, desired clinical outcomes and QoL could be achieved. The management of older and frail patients with kidney failure should embrace a multidisciplinary approach involving doctors from various specialties, nurses, psychologists, dieticians and physiotherapists, as well as caregivers and family members. To this end, additional training in SDM for healthcare professionals could be essential in order to equip them with the necessary skills for effectively communicating with the patient. Furthermore, the establishment of multidisciplinary ‘low GFR’ clinics specifically designed for the elderly could serve as a dedicated resource to address the unique challenges faced by the elderly population with kidney failure.

## Supplementary Material

gfad211_Supplemental_File

## Data Availability

The data underlying this article will be shared on reasonable request to the corresponding author.
