# Supplementary material for: Shared decision making in elderly patients with kidney failure
Source: Nephrol Dial Transplant. 2023 Sep 23;39(5):742–51. doi: 10.1093/ndt/gfad211 (PMC11045282; doi:10.1093/ndt/gfad211)
Supplement: gfad211_Supplemental_File [file gfad211_supplemental_file.docx]

**Supporting shared decision making in elderly patients with kidney failure matching evidence with patient preferences: a narrative review**

**^1^Mehmet Kanbay*, ^2^Carlo Basile*, ^3,4^Yuri Battaglia, ^5^Alessandro Mantovani, ^1^Furkan Yavuz, ^6^Francesco Pizzarelli, ^7^Valerie A. Luyckx, ^8^Adrian Covic, ^9^Vassilios Liakopoulos, ^10^Sandip Mitra** on behalf of the EuDial Working Group of ERA

* **These authors contributed equally to this work**

Corresponding author:

Carlo Basile, MD

Via Battisti 192

74121 Taranto - ITALY

Tel + 39-099-4773688

Mobile + 39-3290628486

E-mail: [basile.miulli@libero.it](mailto:basile.miulli@libero.it)

**SUPPLEMENTARY MATERIAL**

**Table 1S.** Synthesis of the main findings of the most relevant studies comparing dialysis with CKM in elderly patients.

| **Authors,**  **Reference number** | **Study Design** | **Total number (%male)** | **Age (years)** | **Study characteristics** | **Main results** |
| --- | --- | --- | --- | --- | --- |
| Carson et al.  (15) | RS | 202, (67.5) | - RRT group (mean): 76.4 - MCM group (mean): 81.6 | - > 70 years - RRT started in a 6-year period - RRT group n=173 - MCM group n=29 | - Median survival 37.8 months for RRT patients and 13.9 months for MCM patients (p < 0.01) - Higher rates of hospitalization of the RRT group compared with the MCM group - MCM group more likely to die at home or in a hospice |
| van Loon et al.  (16) | PS | 281, (64) | - Dialysis group (mean): 75 - MCM group (mean): 82 | - > 65 years - Dialysis group (n = 192): patients were included if < 3 weeks before and < 2 weeks after the first dialysis session. If dialysis would start > 3 weeks after the assessment, patients were excluded - MCM group (n = 89): patients were included if < 3 months after the decision not to start dialysis had been taken and if eGFR < 15 ml/min | - After 6 months, EQ-5D Index did not improve significantly in the dialysis group - Hospitalization occurred in 50% of dialysis group *vs*. 24% of the MCM group (p < 0.01) - In patients older than 80 years, no survival benefit for the dialysis group *vs.* the MCM group |
| Raman et al.  (17) | PS | 204, (62.2) | - Dialysis group (mean): 78.9 - CC group (mean): 83.7 | - >75 years - eGFR <15 mL/min/1.73 m^2^ - Exclusion criteria: NYHA class 3 or 4, heart failure, previous cardiac arrest, solid organ malignancy, Karnofsky performance score < 60, dementia, dialysis only for AKI, ESKD as an emergency admission, planned pre-emptive live donor transplant - Dialysis group n=123 (n=89 on HD, n=34 on PD) - CC group n=81 | - 115 patients ended up with eGFR < 10 ml/min/1.73 m^2^ (73 in the dialysis group, 42 in the CC group) - Median survival starting from eGFR < 15 ml/min/1.73 m^2^ was 42 months for the dialysis group and 31 months for the CC group. The aHR for death in the dialysis group compared to CC was 0.61 (p = 0.01) - Median survival starting from eGFR <10 ml/min/1.73 m^2^ was 36 months for the dialysis group and 12 months for the CC group. The aHR for death in the dialysis group compared to CC was 0.36 (p < 0.001) |
| Brown et al.  (18) | PS | 467, (63) | - Conservative care (mean): 82 - Pre-dialysis (mean): 67 | Inclusion criteria:   - Patients with CKD (stages 4, 5) - With a planned dialysis pathway - With a planned non-dialysis pathway/conservative care - Commenced dialysis during this time period without attending the pre-dialysis clinic either due to late presentation with ESKD - Pre dialysis = 273; conservative care n=122; n=151 underwent dialysis (n = 100 on HD and n = 51 on PD) | - Greater survival in the pre-dialysis group both for those who required dialysis (hazard ratio [HR] for death, 0.30; p = 0.003) and for those who did not require dialysis (HR for death, 0.23; p < 0.001) - No statistically significant survival difference between all dialysis patients (n=18) and the conservative care group (n=52) when the analysis was restricted to patients aged >75 years and >2 comorbidities (at least one is CHF or IHD) (HR for death 0.48; p = 0.08) |
| Murtagh et al.  (20) | RS | 129, (65) | - CKM group   75-79 years n=12  80-84 years n=36  85-89 years n=24  >89 years n=5   - Dialysis group   75-79 years n=24  80-84 years n=23  85-89 years n=5  >89 years n=0 | - >75 years - Received multidisciplinary pre-dialysis care - Who had chosen dialysis or CKM | - 1 and 2-year survival rates 84% and 76% in the dialysis group (n=52) and 68% and 47% in the conservative group (n=77), with significantly different cumulative survival (log rank 13.6, p < 0.001) - In patients with high comorbidity, the survival advantage offered by dialysis was no longer evident (log rank <0.001, df 1, p = 0.98) |
| Tam-Tham et al.  (21) | RS | 838, (48.6) | - All groups (mean): 79.1 - Dialysis group (mean): 76.3 - Non-dialysis group (mean): 83.2 | - $\geq$65 years - eGFR <10 ml/min/1.73 m^2^ - Exclusion criteria: dialysis initiated on or before the index date, died on the index date, with a kidney transplant | - Significantly lower risk of death of dialysis patients for the first 3 years of follow-up (HR 0.59), but not thereafter (HR 1.22) - Dialysis patients had a higher risk of hospitalization (HR 1.40) |
| Shah et al.  (24) | PS, CS | 129, (65) | - Dialysis group (median): 81 - CKM group (median): 83 | - >75 years - ESKD managed with dialysis - With eGFR <10 ml/min/1.73 m^2^ managed with CKM - Exclusion criteria: cognitive impairment, patients unable to read English and patients who were legally blind | Dialysis group had:   - Lower mean SF-6D utility (−0.05) - Lower KDQoL Physical Component Summary score (−3.17) - Lower Mental Component Summary score (−2.41) - Lower quality of life due to burden (−28.59) - Lesser symptoms (−5.93) - Lesser effects of kidney disease (−16.49) - Lower overall ICECAP-O well-being (−0.07) |
| So et al.  (25) | RS | 604, (60) | - CKM group (median): 82 - HD group (median): 69 - PD group (median): 69 - Kidney transplant group (median): 57 | - All patients in CKM and RRT had > 1 questionnaire in the 6-year period. - CKM group n=144 - HD group n=265 - PD group n=59 | - Median survival time in the CKM group was 14.1 months, significantly shorter compared with the other groups (p < 0.001) - At first, CKM group had more problems with mobility, self-care, and ability to perform usual activities. Pain/ discomfort and anxiety/depression were not higher - In the CKM group there was no significant decline in mobility, self-care, ability to perform their usual activities, pain/discomfort, or anxiety/depression after 12 months or in QoL scores after 18 months compared with the other groups |
| Kurella Tamura et al.  (26) | PS | 212, (50.9) | - Overall age (mean): 64 - No transition to dialysis (mean): 64.7 - Transitioned to dialysis after baseline (mean): 64.5 - Transitioned to dialysis before baseline (mean): 62 | - eGFR <20 ml/min/1.73 m^2^ - eGFR projected to decrease to <20 ml/min/1.73 m^2^ over the subsequent year based on a CRIC prediction model - Or dialysis initiation within the past two years - Exclusion criteria: coexisting disease likely to affect survival, previous treatment with dialysis or kidney transplant, residence in nursing homes, or inability to provide consent - No transition to dialysis n=123 - Transitioned to dialysis after baseline n=37 - Transitioned to dialysis before baseline n=52 | - The estimated net difference in cognitive z scores at 2 years for participants who transitioned to dialysis compared to participants who did not transition to dialysis was –0.01 for global cognition, –0.24 for memory, and –0.33 for executive function - Dialysis initiation was associated with loss of executive function with no change in other aspects of cognition |

PS: prospective, RS: retrospective, CS: cross-sectional, CHF: congestive heart failure, IHD: ischemic heart disease, CKM: conservative kidney management, MCM: maximum conservative management, CC: conservative care, QoL: quality of life, SF-6D: short-form six dimensions, KDQoL: kidney disease quality of life, ICECAP-O: Investigating Choice Experiments Capability Measure, NYHA: New York Heart Association.

**Table 2S.** Synthesis of the main findings of the most relevant studies comparing PD with HD in elderly patients.

| **Authors,**  **Reference number** | **Study Design** | **Total number, (%male)** | **Age (years)** | **Study characteristics** | **Main results** |
| --- | --- | --- | --- | --- | --- |
| Parapiboon et al.  (39) | RCT | 207, (50) | - Urgent start HD (mean): 55   Urgent start PD (mean): 57 | - >18 years - eGFR <15 ml/min for > 3 months - Accepted long-term dialysis and required immediate dialysis treatment without access to definitive dialysis - Exclusion criteria: medical or social contraindications to PD, life-threatening CKD complications requiring urgent start of dialysis, hemodynamic instability, Karnofsky performance status < 40, terminal illness - Urgent start HD n=103   Urgent start PD n=104 | - Urgent-start PD group had a lower composite complication rate at 6 weeks (19% *vs.* 37%, RR 0.52), with a reduction in dialysis-related complications (4% *vs*. 24%, RR 0.16) - No significant differences between the 2 groups with respect to patient and technique survival rates at 1 and 6 weeks |
| Winkelmayer et al.  (40) | RS | 2503, (56) | - HD group:   66-74 years n=981  75-84 years n=838  >85 years n=147   - PD group:   66-74 years n=277  75-84 years n=225  >85 years n=35 | - >65 years - Members of Medicare or Medicaid for at least 12 months - Kidney failure with at least 1 month of dialysis - HD group n=1966 - PD group n=537 | - In the first 90 days after dialysis initiation, PD group had a higher mortality rate (HR 1.16) - After 90–180 days, there were no differences between PD and HD (HR 1.03) - Between 180-365 days, PD had a higher mortality rate (HR 1.45) - In a propensity score-matched paired analysis, 1-year survival rate was 43% on PD *vs*. 51% on HD |
| Termorshuizen et al.  (41) | PS | 1222, (60) | - HD group (mean): 62.3 - PD group (mean): 52.1 | - >18 years old - Begin dialysis as the first RRT, survived first 3 months of dialysis - Exclusion criteria: late referral to the nephrologist, early modality switching, AKI - HD group n=742   <45 years n=89  45-60 years n=187  60-70 years n=203  >70 years n=263   - PD group n=480   <45 years n=147  45-60 years n=173  60-70 years n=100  >70 years n=60 | - No statistically significant differences in adjusted mortality rates between HD and PD patients in the first 2 years - In the years thereafter, increases in mortality rates for PD patients and resulting decreases in RR in favor of HD (*e.g.*, months 24 - 36, adjusted RR 0.53) |
| Wong et al.  (42) | RS | 1376, (61) | - Traditional cohort   HD group (mean): 66.8  PD group (mean): 64.8   - Eligible cohort   HD group (mean): 65.3  PD group (mean): 64.7   - Eligible outpatient cohort   HD group (mean): 64.1  PD group (mean): 64.1 | - >18 years old - Had ESKD, received at least 1 outpatient dialysis, completed a multidisciplinary modality assessment - Exclusion criteria: previous kidney transplant, recovery of kidney function within 180 days of dialysis start - Traditional cohort (all patients completed modality assessment, regardless of their eligibility for PD, to mirror the population used in traditional analyses)   HD group n=1579  PD group n=453   - Eligible cohort (patients eligible for both dialysis modalities to reflect those faced with a modality choice in clinical practice)   HD group n=926  PD group n=450   - Eligible outpatient cohort (patients eligible for both modalities who initiated dialysis therapy electively as outpatients to determine whether exclusion of patients who initiated dialysis therapy in the hospital affected results)   HD group n=465  PD group n=409 | - Traditional cohort   -No statistically significant difference in all-cause mortality between HD and PD in the elderly:  -Patients < 65 years: PD was associated with significantly lower risk for death when compared to HD in the first 3 years of dialysis therapy (aHR PD:HD 0.60)   - Eligible cohort   -The effect of dialysis modality on survival did not vary over time; PD and HD were associated with a similar risk for death (aHR PD:HD 1.08)   - Eligible outpatient cohort   People treated with PD and HD had similar risks for all-cause mortality (aHR PD:HD 1.19) |
| Brown et al.  (43) | CS | 140, (70) | - HD group (mean): 73.4 - PD group (mean): 73.1 | - >65 years old - On dialysis for a minimum of 90 days - Had not been hospitalized for 30 days - Exclusion criteria: clinically obvious cognitive impairment, life expectancy < 6 months - HD group n= 70   65-69 years n=21  70-79 years n=41  80-89 years n=8   - PD group n=70   65-69 years n=21  70-79 years n=41   - 80-89 years n=8 | - PD group had better SF-12 MCS scores (p=0.046) with significantly less possible depression (p =0.015) and illness intrusion (p =0.006) - Total number of symptoms was also significantly lower in PD patients (8.6 and 9.7, respectively, p = 0.039) - HD patients experienced greater intrusion of the illness and/or their treatment in relation to their health (p = 0.001) and diet (p ≤ 0.0001) compared to PD group - Patients on dialysis >12 months: HD patients showed significantly more illness intrusion (p = 0.005), higher depression scores (p = 0.008) and worse SF-12 MCS scores (p = 0.013) than their PD equivalents |
| Iyasere et al.  (45) | CS | 251, (60) | - HD group (median): 75   aPD group (median): 76 | - >60 years - aPD with paid health-care workers or family members - Patients on HD required hospital transport to attend dialysis sessions - On dialysis for > 3 months - Free from hospitalization for >30 days - Exclusion criteria: known cognitive impairment, unable to understand English, life expectancy of < 6 months - HD group n=122   aPD group n=129 | - Patients on aPD had a higher prevalence of depression (HADS>8; PD=38.8%; HD=23.8%; p = 0.05) and higher HADS depression score (median: PD=6; HD=5; p=0.05) but higher RTSQ scores (median: PD=55; HD=51; p < 0.01) - In a generalized linear regression model adjusting for age, sex, comorbidity, dialysis vintage, and frailty, aPD was still associated with higher RTSQ scores (p=0.04) but not with other QoL measures |
| Zeng et al.  (49) | CS | 265, (47.5) | - HD group (mean): 53.9 - PD group (mean): 58.3 | - ≥18 years - ESKD patients on HD or PD for > 3 months - Ability to read and complete questionnaires - With complete clinical data - HD group n=115 - PD group n=150 | - PD group had lower scores on the Montreal Cognitive Assessment than HD group (β=−8.35), 36-Item Short Form Survey (β=−10.20), and kidney disease-related quality of life assessment (β=−8.67) |
| Harris et al.  (50) | PS | 174, (66) | - HD group (mean): 77 - PD group (mean): 76.8 | - >70 years old - On dialysis for at least 90 days - Exclusion criteria: terminal illness with a life expectancy < 6 months, diagnosis of psychosis, cognitive impairment - New patients n=78 (recruited after 90 days of chronic dialysis)   PD group n=36  HD group n=42   - Stock patients n=96 (already on chronic dialysis during the recruitment period)   PD group n=42  HD group n=54 | - Annual mortality (deaths/100 person–years): 26.1 in PD patients; 26.4 in HD patients - Hospitalization rates (admissions/person–year): 1.9 in PD patients; 2.0 in HD patients - Adjusted relative risks showed no effect of modality on clinical outcomes - Similar SF-36 scores between PD and HD patients, but higher KDQoL scores in PD patients (3.5 points higher) - However, no effect of dialysis modality on QoL at 6 or 12 months |
| Farragher et al.  (53) | PS | 236, (56) | - HD group (mean): 74.7 - PD group (mean): 73.2 | - >65 years - Undergoing PD or HD in a specific 1-year period - HD group n=162 - PD group n=74 | - 40 PD patients (54%) experienced 86 falls while 76 HD patients (47%) experienced a total of 305 falls (crude fall rate 1.25 *vs*. 1.60 respectively, OR falls in PD patients 0.78, p = 0.04) - After adjustment for differences in comorbidity, number of medications, and other demographic differences, PD patients were no less likely to experience accidental falls than HD patients (OR 1.63, p = 0.1) |
| Saka et al.  (55) | RS | 56, (68) | - HD group (mean): 84.8 - PD group (mean): 86.2 | - >80 years - Started dialysis in a 2-year period - Exclusion criteria: not discharged since dialysis initiation and died - HD group n=42 - PD group n=14 | - Mortality was higher in PD group than in HD group (p = 0.003) - Long-term hospitalization (>180 days) only in HD patients (PD *vs*. HD: 0.0 *vs.* 16.7%; p = 0.102) - In patients with Barthel index scores <100, the long-term hospitalization difference was significant (PD vs. HD: 0.0 *vs.* 30.4%; p = 0.04) - 6 of the 7 deceased PD patients and 1 of the 10 deceased HD patients died at home (p = 0.002) |

PS: prospective, RS: retrospective, CS: cross-sectional, RCT: randomized clinical trial, aPD: assisted PD, HADS: Hospital Anxiety and Depression Scale, RTSQ: Renal Treatment Satisfaction Questionnaire, AKI: acute kidney injury.

**Table 3S.** Synthesis of the main findings of the most relevant studies investigating the outcomes of different VA types in elderly patients.

| **Authors,**  **Reference number** | **Study design** | **Total number, (%male)** | **Age (years)** | **Study characteristics** | **Main results** |
| --- | --- | --- | --- | --- | --- |
| Drouven et al.  (66) | PS | 694, (59) | - Octogenarians (>80 years) (mean): 82.1 - Control group (<80 years) (mean): 61.7 | - Patients with chronic kidney dysfunction requiring HD in a specific 3-year period - Octogenarians:   RC AVF n=19  BC AVF n=36  AVG n=8  BVT n=2   - Control group:   RC AVF n=203  BC AVF n=248  AVG n=124  BVT n=54 | - No significant differences in patient survival, with 5-year survival rate of 63.8% (65.9%) in the octogenarian group and 57.2% (62.2%) in the control group (p = 0.866) - In the octogenarian group, primary failure rate was higher in the RC AVFs: 42.1% (p = 0.006). - BC AVF had improved assisted patency compared with the other VAs in the octogenarians (p = 0.016) - In the adjusted analysis, BC AVF was associated with an increase in primary patency (HR 0.70; p = 0.006) and primary assisted patency (HR 0.58; p = 0.006) compared with other VAs |
| Jhee et al.  (67) | RS | 23100, (56.1) | - Overall (mean): 73.7 - AVF group (mean): 73 - AVG group (mean): 74.3 - CVC group (mean): 75.9 | - >65 years - From a nationwide registry - With ESKD in a specific 17-year time period - Exclusion criteria: insufficient data or no data on VA | - AVFs showed the best survival rates, CVCs the worst ones - AVG showed a greater risk for all-cause mortality only in patients in the highest sCCI group (HR 1.33; p = 0.04) - AVGs in the highest sCCI tertile were associated with 75% increased risk for all-cause mortality compared with AVFs in the lowest sCCI (HR 1.75; p = 0.004) - AVGs in the highest sCCI group showed a significantly increased risk of hospitalization due to access malfunction (OR 1.74; p = 0.03). |
| Arhuidese et al.  (68) | RS | 78341, (44) | - <50 years group n=10150 - 50-59 years group n=13167 - 60-69 years group n=19975 - 70-79 years group n=20307 - >80 years group n=14742 | - All patients in the U.S. Renal Data System (USRDS) initiating HD in a 7-year period | - Primary patency at 5 years comparing: a. <50 years; b. 50 - 59 years; c. 60 - 69 years; d. 70 -79 years: e. 80 years. It was 12% in a; 12% in b; 9% in c; 9% in d; 8% in e (p < 0.001) - Primary assisted patency at 5 years was 20% in a; 21% in b; 18% in c; 17% in d; 14% in e (p < 0.001) - Secondary patency at 5 years was 36% in a; 39% in b; 36% in c; 30% in d; 31% in e (p < 0.001) - No significant difference in primary patency (aHR, 1.00; p < 0.001), primary assisted patency (aHR, 1.00; p < .001), and secondary patency (aHR, 1.00; p = 0.029) with increasing age - Decrease in severe prosthetic graft infection requiring graft excision (aHR, 0.99; p < 0.001) and increase in mortality (aHR, 1.03; p < 0.001) for the older age categories |
| Grubbs et al.  (69) | RS | 117277, (53.7) | - Overall (mean): 76.4 - AVF (mean): 75.8 - AVG (mean): 76.2 - CVC + AVF (mean): 75.8 - CVC + AVG (mean): 76 - CVC only (mean): 76.6 | - AVF n= 15653 - AVG n=5455 - CVC + AVF n=17556 - CVC + AVG n=4073 - CVC only n=74540 | - CVC only group had more limited functional status (25.5 *vs.* 10.8% in AVF group) and more prior hospital days than those with AVF (mean 18.0 *vs*. 5.4) - In the unadjusted model, the likelihood of death was higher for AVG group (HR 1.20), CVC + AVF (HR 1.34), CVC + AVG (HR 1.46) and CVC only (HR 1.95), compared with AVF (p < 0.001) - After adjusting for the usual covariates, the association attenuated: −23.7% overall (AVF *vs.* all other access types). The HR of AVG was 1.21, the HR of CVC + AVF was 1.27; the HR of CVC + AVG 1.38 and the HR of CVC only was 1.69 |
| Lee et al.  (72) | RS | 9458, (52) | - Overall (mean): 77 - AVF (mean): 77 - AVG (mean): 78 | - >67 years - Exclusion criteria: using AVF or AVG or a maturing AVF or AVG at HD initiation - Patients underwent AVF or AVG surgery in the 2-year period preceding HD initiation - AVF group n=7433 - AVG groupn=2025 | - CVC dependence was greater in every month during the 6-month period after AVF *vs.* AVG placement (p < 0.001) - Rates of all-cause infection-related hospitalization (aRR 0.93; P=0.01) and bacteremia/septicemia-related hospitalization (aRR 0.90; P=0.02) were lower in the AVF *vs*. AVG group as was the adjusted risk of death (HR 0.76; P<0.001) |
| Liu et al.  (73) | RS | 688, (62) | - Non-elderly (n=418) (mean): 53 - Elderly (n=184) (mean): 69.4 - Older (n=184) (mean): 78.7 | - ESKD patients initiated maintenance HD in a 2-year period - Exclusion criteria: transition from PD or failed kidney transplant, on long-term CVC and with AVG as a vascular access. - Non-elderly n=418: RC (n=314), BC (n=92), BB (n=12) - Elderly n=184: RC (n=116), BC (n=64), BB (n=4) - Older n=86: RC (n=50), BC (n=29), BB (n=7) | - The outcomes of vascular accesses were comparable in the 3 age groups with similar proportions of functional AVFs (80.4% *vs.* 79.3% *vs*. 75.6%, p = 0.832) and comparable 1-, 3- and 5-year primary and secondary patency rates (p = 0.351 and 0.282, respectively) - Longer maturation time (2.78 *vs.* 2.86 *vs*. 3.72 months, p = 0.01); a higher mean number of interventions to assist maturation of the first AVF were required in older patients (0.19 *vs*. 0.22 *vs.* 0.35, p = 0.014) - After AVF creation, median patient survival in the non-elderly, elderly and older patients was 65.2 *vs*. 55.1 *vs*. 49.8 months, respectively |
| Beaulieu et al.  (74) | RS | 524, (63) | - >75 years group (mean): 81 - 65-75 years group (mean): 71 - <65 years group (mean): 52 | - Patients who had their first AVF creation in a specific center in a 5-year period - Both pre-dialysis and dialysis patients - Exclusion criteria: death, start on PD, kidney transplant within 3 months of AVF creation - >75 years group n=147 - 65-75 years group n=171 - <65 years group n=206 | - No differences in rates of primary failure, loss of primary patency, complications, or need for intervention among the three age groups - Significant association of age with secondary patency and functional patency, with age > 75 years being an independent risk factor for shortened lifespan of the fistula - For patients aged >75 years, secondary patency at 3 years was 64% compared to 75%-78% for younger patients - Functional patency at 2 years was 69% for those aged >75 years compared to 78%-81% for younger patients. |
| Diandra et al.  (75) | RS | 47, (57) | - Octogenerians (>80 years) (mean): 83 | - >80 years old - Undergoing AVF creation in a specific 6-year time period - CVCs   CVC in situ n=35  CVC ipsilateral to AVF n=10   - AVFs   RC AVF n=15  BC AVF n=30  BBT AVF n=2 | - At 12 months, primary patency rate 15% (n=7), assisted primary patency rate 28% (n=13), primary failure rate 72% (n=34) - Early thrombosis (<30 days) rate 9% (n=4). Subgroup analysis showed assisted primary patency in 20% of RC AVFs (3 of 15) and 33% of BC AVFs (10 of 30) - No patients had wound site post-operative infection. In the follow-up of 24 months, 31% (n=11) were admitted with CVC-related sepsis - Survival rates at 12, 24, 36, 48, and 60 months from the time of AVF creation: 86%, 73%, 63%, 57%, and 52%, respectively |
| Hwang et al.  (76) | RS | 594, (62) | - Older group (>70 years old) (mean): 75.9 - Younger group (<70 years old) (mean): 55.3 | - ESKD patients undergoing VA creation in a specific 3-year period - Older group n=193   AVF n=130  AVG n=63   - Younger group n=401   AVF n=293  AVG n=108 | - AVFs: primary success rate 83.6% in the older group *vs.* 94.3% in the younger group (p = 0.001); overall primary patency at 6 and 12 months (73.1% and 57.1%, respectively, in the older group *vs.* 86.7% and 77.7%, respectively, in the younger group; p = 0.009) - No differences in the primary success rate and primary patency rates for AVGs - Primary success rate of the upper arm not different between the two groups (94% *vs.* 97%; p = 0.425) |
| Misskey et al.  (78) | RS | 941, (62) | - >80 years group n=152 - 65-79 years group n=397 - <65 years group n=392 | - All patients with a first-time autogenous AV access in either arm for HD - Exclusion criteria: subsequent fistula creation attempts on the ipsilateral arm, tunneled CVCs, and AVGs - RC: >80 years n=48; 65-79 years n=141; <65 years n=140 - BC: >80 years n=78; 65-79 years n=170; <65 years n=149 | - Cumulative primary patency at 36 months: 14±5% (>80 years) *vs* 26±3% (<65 years), p<0.006 - Cumulative secondary patency at 36 months: 54±5% (>80 years) *vs.* 72±3% (<65yo), p<0.002 - RC AVF secondary patency at 36 months: 41±8% (>80 years) *vs.* 68±4% (< 65 years), p<0.013 - No statistical difference in RC AVF primary patency and BC AVF primary and secondary patency among the three age groups |
| Cui et al.  (82) | RS | 182, (63.2) | - AVF group:   75-59 years n=63  80-84 years n=42  >85 years n=33   - AVG group:   75-59 years n=19  80-84 years n=15  >85 years n=10 | - >75 years old - All HD access procedures performed in a 3-year period | - Primary failure rate higher for AVFs than for AVGs (OR 2.89; p = 0.008) - More AVFs required one or more interventions before their successful use compared with AVGs (31% *vs*. 10%; p = 0.03) - Time to CVC-free dialysis longer for AVFs than for AVGs (p < 0.001) |
| Zhang et al.  (85) | RS | 39721, (60) | - Overall   AV-access group (mean): 68  CVC group (mean): 68 | - >18 years old - Registered in a register and HD start as their first form of RRT in a specific 10-year period - Exclusion criteria: no documentation for initial VA type - <65 years group n=16655   AV-access n=3466  CVC n=13189   - 65-74 years group n=10738   AV-access n=2376  CVC n=8362   - 75-85 years group n=10271   AV-access n=2066  CVC n=8205   - >85 years group n=2057   AV-access n=311  CVC n=1746 | - AV-access use associated with lower adjusted mortality compared with CVC use in each age category (HR 0.67; 0.76; 0.77; 0.73, respectively) |
| Jakes et al.  (86) | RS | 167, (61) | - Overall (mean): 83.3 - CVC (mean): 82.9 - AVF (mean): 81.8 | - >80 years old - HD start in a specific 11-year period - Exclusion criteria: emergency dialysis, AVGs, kidney transplant - CVC group n=101 - AVF group n=25 - CKM group n=41 | - Mean age of CKM patients reaching an eGFR < 10ml/min/1.73 m^2^ 85.8 years - Mean (median) survival on dialysis 2.2 (1.8) years for AVF patients, 2.1 (1.2) for CVC patients, and 1.5 (0.9) for CKM patients (p = 0.107, controlling for age/sex p = 0.519) |
| Hicks et al.  (87) | RS | 507791, (57) | - AVF group (mean): 64.1 - AVG group (mean): 65.5 - CVC group (mean): 63.2 | - >18 years old - HD start in a specific 4-year period - Exclusion criteria: missing data related to age or initial dialysis VA type, dialysis start before 2006, previous kidney transplant, death within 90 days of initiating dialysis - AVF group n=71316 - AVG group n=17543 - CVC group n=418932 | - Older age was a significant predictor of overall mortality (aHR 1.03; p < 0.001). Compared with patients with CVC, overall risk-adjusted mortality was lowest in patients with AVFs (aHR 0.63; p < 0.001) followed by AVGs (aHR 0.83; p < 0.001) - AVF superior to both CVC and AVG for all age groups (p < 0.001) - No significant differences when comparing adjusted mortality of AVGs and CVCs in patients aged 18-48 years or in patients aged more than 89 years; AVGs superior to CVCs in patients 49-89 years of age (aHR 0.811; p < 0.001) - Mortality benefit of AVFs consistently superior to that of AVGs and CVCs in patients of all ages (all, p < 0.001) - AVFs superior to AVGs and CVCs regardless of the patient’s age, including octogenarians. In contrast, the mortality benefit of AVGs *vs.* CVCs may not apply to younger (18-48 years) or older (> 89 years) age groups |
| Arhuidese et al.  (88) | RS | 124421, (54.8) | - AVF at start of dialysis (mean): 82 - AVG at start of dialysis (mean): 82.2 - AVF converted (mean): 81.7 - AVG converted (mean): 82.3 - Permanent CVC (mean) 82.3 | - >75 years - With AVF, AVG, or CVC for HD start - Exclusion criteria: patients with a pre-emptive AV access but did not progress to ESKD - AVF at start of dialysis n=19173 - AVG at start of dialysis n=4840 - AVF converted=29872 - AVG converted n=10712 - Permanent CVC n=59824 | - Compared with AVF initiates, relative mortality was significantly higher for AVG initiates (aHR 1.24; p < 0.001), AVF converts (aHR 1.36; p < 0.001), AVG converts (aHR 1.62; p < 0.001), and CVC-persistent patients (aHR 2.23; p < 0.001) - Primary patency (aHR 1.21; p < 0.001) and primary assisted patency (aHR 1.31; p < 0.001) higher for AVFs - Secondary patency was higher for AVGs within the first 4 months (aHR 1.12; p < 0.001), but higher for AVFs beyond that time point (aHR 1.25; p < 0.001) - Maturation rate and median time to maturation were 80% *vs.* 84% (p < 0.001) and 46 *vs.* 26 days (p < 0.001) for AVFs *v*s. AVGs |
| DeSilva et al.  (89) | RS | 115425, (52.9) | - Overall (mean):76.9 - AVF (mean): 76.2 - AVG (mean): 76.2 - CVC (mean): 77.2 | - >67 years - Incident HD patients - With confirmed first pre-dialysis access placement - AVF n=21436 - AVG n=3472 - CVC n=90517 | - Significantly inferior survival of CVC group compared to AVF (HR 1.77; p < 0.001) - No significant mortality difference between patients with AVG and AVF (HR 1.05; p = 0.06) - Analyzing mortality stratified by age groups, AVGs had inferior mortality outcomes compared with AVFs for the 67-79 years age group (HR 1.10; p = 0.007), but in 80-89 and the > 90 years age groups there were no significant differences |
| Kim et al.  (90) | RS | 2045, (59) | - Overall (mean): 58.5 - Percutaneous catheter (mean): 59 - Tunneled cuffed catheter (mean): 58.6 - AV access (mean): 59.4 | - Diagnosed with AKI or CKD and undergoing HD for > 3 months - Exclusion criteria: death in the first 3 months after initiation of HD, no further VA, kidney transplant, conversion to PD or CVC   Percutaneous catheter n=815  Tunneled cuffed catheter n=491   - AV access (AVF, AVG) n=739 | - Survival rates of patients who received a CVC, aged 65-74 years and ≥ 75 years were reduced, but not for < 65 years (log-rank test; p < 0.001, p = 0.007, and p = 0.278) - After adjusting for confounding factors, patients less than 65 years old, percutaneous and tunneled cuffed catheters, were not associated with 5-year mortality ([HR 1.28 and 1.04, respectively) - Patients aged 65 to 74 and ≥ 75 years, percutaneous catheters ([HR 1.97 and 1.93, respectively) and tunneled cuffed catheters ([HR 1.89 and 1.34, respectively) were significantly associated with 5-year mortality |

PS: prospective, RS: retrospective, AV: arteriovenous, AVF: arteriovenous fistula, AVG: arteriovenous graft, CVC: central venous catheter, RC: radiocephalic, BC: brachiocephalic, BB: brachiobasilic, AKI: acute kidney injury, BVT: basilic vein transposition, sCCI: Simplified Charlson comorbidity index.
